# Evaluation of a civic engagement approach to catalyze built environment change and promote healthy eating and physical activity among rural residents: a cluster (community) randomized controlled trial

**DOI:** 10.1186/s12889-022-13653-4

**Published:** 2022-09-04

**Authors:** Rebecca A. Seguin-Fowler, Karla L. Hanson, Deyaun Villarreal, Chad D. Rethorst, Priscilla Ayine, Sara C. Folta, Jay E. Maddock, Megan S. Patterson, Grace A. Marshall, Leah C. Volpe, Galen D. Eldridge, Meghan Kershaw, Vi Luong, Hua Wang, Don Kenkel

**Affiliations:** 1 Institute for Advancing Health Through Agriculture, Texas A&M AgriLife, College Station, TX 77843 USA; 2 Department of Public and Ecosystem Health, Cornell University, Ithaca, NY 14853 USA; 3 Institute for Advancing Health Through Agriculture, Texas A&M AgriLife Dallas Center, Dallas, TX 75252 USA; 4 Friedman School of Nutrition Science and Policy, Tufts University, Boston, MA 02155 USA; 5 School of Public Health, Texas A&M University, College Station, TX 77843 USA; 6 Jeb E. Brooks School of Public Policy, Cornell University, Ithaca, NY 14853 USA

**Keywords:** Civic engagement, Built environment, Nutrition, Physical activity, Rural health equity, Social influence

## Abstract

**Background:**

Prior studies demonstrate associations between risk factors for obesity and related chronic diseases (e.g., cardiovascular disease) and features of the built environment. This is particularly true for rural populations, who have higher rates of obesity, cancer, and other chronic diseases than urban residents. There is also evidence linking health behaviors and outcomes to social factors such as social support, opposition, and norms. Thus, overlapping social networks that have a high degree of social capital and community cohesion, such as those found in rural communities, may be effective targets for introducing and maintaining healthy behaviors.

**Methods:**

This study will evaluate the effectiveness of the Change Club (CC) intervention, a civic engagement intervention for built environment change to improve health behaviors and outcomes for residents of rural communities. The CC intervention provides small groups of community residents (approximately 10–14 people) with nutrition and physical activity lessons and stepwise built environment change planning workshops delivered by trained extension educators via in-person, virtual, or hybrid methods. We will conduct process, multilevel outcome, and cost evaluations of implementation of the CC intervention in a cluster randomized controlled trial in 10 communities across two states using a two-arm parallel design. Change in the primary outcome, American Heart Association’s Life’s Simple 7 composite cardiovascular health score, will be evaluated among CC members, their friends and family members, and other community residents and compared to comparable samples in control communities. We will also evaluate changes at the social/collective level (e.g., social cohesion, social trust) and examine costs as well as barriers and facilitators to implementation.

**Discussion:**

Our central hypothesis is the CC intervention will improve health behaviors and outcomes among engaged citizens and their family and friends within 24 months. Furthermore, we hypothesize that positive changes will catalyze critical steps in the pathway to improving longer-term health among community residents through improved healthy eating and physical activity opportunities. This study also represents a unique opportunity to evaluate process and cost-related data, which will provide key insights into the viability of this approach for widespread dissemination.

**Trial registration:**

ClinicalTrials.gov: NCT05002660, Registered 12 August 2021.

**Supplementary Information:**

The online version contains supplementary material available at 10.1186/s12889-022-13653-4.

## Background

Nearly 70% of U.S. adults are overweight or obese [1], and with this comes a multitude of consequences, including increased risk for several types of cancer [2, 3], diabetes [4], and cardiovascular disease [5]. Only 20% of US adults meet physical activity (PA) guidelines [6]. Inadequate PA increases risk for many chronic conditions, including some types of cancer, obesity, metabolic syndrome, and hypertension [7]. Adding as little as 10–15 min per day of PA or reducing sedentary time by 0.5 to 1 h per day confers significant health benefits, including improving biomarkers of chronic disease and reducing all-cause mortality risk [8–11]. Likewise, consuming a healthy diet, including adequate amounts of fruits and vegetables (FV), is associated with lower risk of cancer and obesity [12]; yet only about 10% of U.S. adults meet FV intake recommendations [13]. Increasing FV intake by as little as one serving per day significantly decreases all-cause mortality risk [14]. Inadequacies in PA and FV intake are major contributors to healthcare expenditure [15, 16]. This is particularly relevant for rural populations, who tend to have higher rates of cancer, obesity, physical inactivity, and poor diet than urban residents [17–21]. Rural areas also have higher rates of poverty [22] and more limited access to healthcare [23], healthy food [24], PA facilities [25], and active transportation opportunities [26]. Thus, effective and feasible interventions are needed to increase and enhance rural healthy eating and PA opportunities.

Previous evidence has shown an association between built environment features and cancer, obesity, and related health behaviors, including PA and dietary patterns [27–30]. Similarly, changes in built environment features and policies have shown potential to improve health [31–38]. Both the Centers for Disease Control and Prevention and the World Health Organization acknowledge the health impact of the environments in which people interact and recommend making changes to these environments to help people lead healthier lives [39, 40]. In their 2018 report, the National Association of County and City Health Officials suggested integrating support for policy, systems, and environmental interventions that promote health equity in cancer prevention and control planning at the local level [41]. Rural built environments often pose unique challenges, including active transport challenges (e.g., poor pedestrian infrastructure, high speed limits, lack of bike lanes) and long distances to healthy food and PA opportunities [42]. Thus, opportunities to intervene at the built environment and policy levels to encourage healthy eating and active living in rural communities are essential.

Additionally, it is increasingly understood that social environments have an influence on PA and dietary behaviors in a variety of ways, including social support/opposition, norms, and access to resources [43–47]. Yet the influence of social factors such as social capital, community cohesion, and collective efficacy on behavior change in rural populations is inadequately understood. Social networks and norms of self-help and reciprocity are often characterized as positive aspects of rural life [48]. Further, highly connected networks may speed the diffusion of behavioral changes that require strong social reinforcement [49, 50]. On the other hand, in small, isolated communities, entrenched sociocultural norms can limit people’s behavioral choices [51, 52]. Social dynamics are therefore likely to affect outcomes related to policy or built environment changes. Some studies in rural areas have focused on social-environmental determinants of health behavior change, highlighting facilitators in the social (e.g., accountability, support) and community (e.g., norms, access) domains and related barriers (e.g., social: family responsibilities, discouragement from others; community: lack of FV access, built environments unconducive to PA) [53–57].

Civic engagement interventions for built environment change, or CEBEC, is an approach that accounts for social contexts and has environmental change as a major focus. It therefore represents a novel and promising approach for promoting behavior change in the rural context. The CEBEC approach rests on civic engagement, defined as “individual and collective actions designed to identify and address issues of public concern” [58]. Civic engagement is inclusive of community volunteerism, which has been linked with positive influences on health behaviors in rural populations [59–68]. In the CEBEC approach groups of citizens are guided through a process of assessing their communities identifying issues and developing and enacting a plan for built environment change. The Change Club (CC) intervention was designed as a CEBEC intervention for rural communities. In this intervention a small group of residents (CC members [CCM]) will work to catalyze change in their community environment relative to food (for example foods in restaurants or schools) or PA opportunities (for example parks or walking trails) by following a stepwise process facilitated by an extension educator.

The theoretical framework for the CEBEC approach rests on Social Cognitive Theory [69] nested within a socioecological framework [70]. At the individual level, civic engagement is designed to promote behavioral skills, including self-regulation, by guiding CCM through a process that includes goal setting and monitoring. It is also designed to positively impact cognitive influences. Self-efficacy may be enhanced since the community project is integrated with diet and PA content that promotes small, achievable changes. At the group level, by identifying and making changes to environmental factors that affect community health, CCM will benefit by gaining a sense of collective efficacy to create cooperative change, which impacts health behaviors [71]. The groups themselves are designed to provide social support, which positively affects health behaviors [72, 73].

The CC intervention is also designed to impact the broader social environment by enhancing bonds of trust and identity as groups work together and with their communities. Because they will choose from a menu of evidence-based community-change strategies, CCM will be able to identify and tailor projects to be reasonably compatible with existing social norms. This is essential for individual- and community-level health behavior change [74], especially in the rural context. There is fairly strong evidence that eating and other health behaviors are transmitted through social networks, via observation/modeling, social rewards, and other mechanisms [75, 76]. It is expected that members of the CCM’s social networks will be impacted as CCM make changes in their own diet and PA behaviors. At the community level, civic engagement provides a potentially powerful way to impact environmental influences on behavior, not just for CCM but also for friends and family members in broader social networks, as well as other community residents who may be impacted by built environment and policy changes. Finally, particularly for CCM, behavior change may be further enhanced via reciprocal determinism, or a positive, reinforcing interaction among behavioral, cognitive, and environmental factors [69].

In previous studies, both rural and urban CEBEC interventions have led to meaningful built environment and policy changes (e.g., allocation of government funds for built environment improvements, sidewalk repair programs, addition of shade trees to encourage walking, and installation of pedestrian flashing light signals) [59–62, 77–81]. However, few studies have evaluated individual-level health behavior or health outcome changes in response to CEBEC projects. Additionally, CEBEC interventions have not been evaluated using well-matched control communities [60, 63–66]. Given the potential of this approach, and current gaps within research to date, there is a need to evaluate rural CEBEC interventions aimed at improving diet and PA. The central hypothesis is that our CEBEC intervention approach, CC, will improve health behaviors and outcomes among engaged residents and their friends and family members, and that these changes can catalyze critical steps in the pathway to improving rural health equity through improved healthy eating and PA opportunities. Thus, the overall objectives of this study are to not only address the knowledge gap but to facilitate built environment change by conducting a cluster randomized controlled trial to test whether or not CC a) improves individual health behaviors by increasing FV consumption and PA opportunities and b) promotes social cohesion and builds social trust among CCM, their friends and family members, and community residents; and to c) examine barriers to implementation and cost and d) examine maintenance of individual and collective changes. Furthermore, our study will facilitate collection of cost data and process evaluation measures to identify effective and cost-effective strategies for dissemination.

### Study aims

#### Aim 1

To evaluate changes in American Heart Association’s Life’s Simple 7 (LS7) composite cardiovascular health score and its components (see Table 3) among residents of CC intervention communities (CCM, friends and family members, and community residents) compared to comparable groups in control communities.

#### Aim 2

To evaluate changes in individual health outcomes (e.g., BMI) and behaviors (e.g., PA levels) as well as adherence to cancer-related recommendations (i.e. World Cancer Research Fund/American Institute for Cancer Research composite score [12]) among residents of CC intervention communities relative to residents of control communities.

#### Aim 3

To evaluate changes at the social/collective level (e.g., social cohesion, social engagement) as well as social network influence on outcomes in CC intervention communities relative to control communities.

#### Aim 4

To examine barriers and facilitators to implementation of the CC including costs and unintended consequences.

#### Aim 5

To examine maintenance of any observed net changes in individual or social/collective measures between CC intervention and control communities.

## Methods

This study will evaluate the effectiveness of the CC intervention in a cluster randomized controlled trial, in which communities are the clusters, using a two-arm parallel design. Cluster randomization was needed because the intervention aims to influence the community environment for healthy eating and PA as well as individual health behaviors and outcomes. We chose a parallel design for statistical efficiency; this is based on the 24-month follow-up data needed to adequately assess CC impacts and the small interclass correlations within towns (0.02–0.04) observed in our previous community randomized studies, which show that the clusters are quite homogenous. Annual longitudinal data will be collected at baseline, + 12, + 24, and + 36 months. Data collected at 24-month follow-up will provide the primary outcome analysis, and data collected at 36 months allow for the examination of maintenance of any observed changes.

### Communities

The study will be carried out in ten paired communities in two states (four in New York and six in Texas). These communities are rural per the Rural–Urban Commuting Area version 2.0 definition [20, 82] and are designated as medically underserved areas and/or Health Professional Shortage Areas [83]. Randomization (based on random numbers computer-generated by research staff) will occur after baseline measurements are collected in both communities within a pair, with five communities starting the CC process and resident-led implementation activities directly after randomization and the remaining five communities serving as controls. It is not feasible to conceal assignment to intervention or control from participants or research staff due to the nature of the design; however, field staff involved in intervention delivery will not be involved in assessing outcomes. At the conclusion of data collection (36 months after baseline), the five control communities will be provided with intervention materials, but their outcomes will not be measured after that time point.

### Participants

The study aims to recruit and enroll 2,260 adults in three inter-related samples in each community: 1) CCMs, 2) CCMs’ friends and family members, and 3) community residents. Extension staff will facilitate the CCs, and in collaboration with the project team, will recruit 10–14 residents to participate in each community’s CC. CCM will be asked to invite friends and family members to participate in the study, and we anticipate a total of 90–112 friends and family members per community to enroll. Approximately 80–100 community residents will also be recruited from each community.

#### Inclusion and Exclusion Criteria

Participants must be at least 18 years of age and English-speaking. Additional eligibility and exclusion criteria for participant groups are shown in Table 1.

**Table 1:** Eligibility and exclusion criteria for each type of participant group

**Participant Group**	**Eligibility Criteria**
Change Club Members	•Provide electronic informed consent •Be willing to be randomized to either group •Score “poor” or "intermediate" on at least one of the American Heart Association’s Life’s Simple 7 composite score items •Live in one of the participating communities in New York or Texas
Friends and Family Members	•Provide electronic informed consent •Be a friend or family member identified by a Change Club Member
Community Residents	•Provide electronic informed consent •Live in one of the participating communities in New York or Texas
Extension Educators	•Provide electronic informed consent •Serve as a Change Club leader
**Participant Group**	**Exclusion Criteria**
All Participants	•Cognitive impairment (if it precludes completion of assessments and/or intervention) •Inability to communicate due to severe, uncorrectable hearing loss or speech disorder (if it precludes completion of assessments and/or intervention) •Severe visual impairment (if it precludes completion of assessments and/or intervention) •Inability to read (as it precludes completion of assessments and/or intervention) •Already included in another study sample (e.g., Community Residents cannot also be Change Club Members)

### Recruitment

CC facilitators will attend community events such as school sporting events, fairs, festivals, community meetings, and other emergent recruitment opportunities, as well as drawing upon their extensive network of community contacts to recruit potential participants. CC facilitators will place flyers and posters at community centers, libraries, restaurants, grocery stores, banks, and other relevant locations. We will utilize zip code mailing lists to mail postcards inviting participation to all adult residents in each community up to three times. Other recruitment efforts will include the use of news releases, social media ads, radio ads, and television ads. Targeted digital advertising methods will be utilized to target our ads using zip codes and relevant keywords. A study website was created to help describe the study in further detail and explain the various roles of participation.

#### CCM recruitment

CCM will complete an online eligibility screener and, if eligible, complete an electronic informed consent process. The local extension educator will also communicate with CCMs to discuss the CC activities.

#### Friends and family members recruitment

CCM will be asked to recruit adults in their ‘social circle’ to complete data collection activities using a unique screening link provided to each CCM. Friends and family members invited by a CCM, if interested, will complete an online eligibility screener and, if eligible, complete an electronic informed consent process.

#### Community resident recruitment

Individuals who screen to be CCM and are deemed ineligible, will be invited to participate as community residents. Community residents will complete an online eligibility screener and, if eligible, complete an electronic informed consent process.

### Intervention

County-level extension agents traditionally provide non-formal education and skill-based learning to adults and children in their communities. A local extension educator in each community will be trained to become a CC facilitator to guide stepwise planning workshops, measure engagement, and guide members through nutrition and PA lessons through in-person, virtual, or hybrid methods. CC facilitators will be trained on the CC curriculum and facilitator guide covering all content modules. Once leaders are trained, they will facilitate the first set of CC modules and continue to meet and support their CC thereafter as needed throughout the study. Table 2 shows the multilevel components and summary of the CC curriculum.

**Table 2:** Summary of change club curriculum

**Theme 1: Fostering Togetherness and Unity**	
** Module 1:** Introduction	Introduction and program overview
** Module 2:** Fostering Engagement	Engaging in community issues
** Module 3:** Team Building	Working effectively as a team
** Module 4:** Assessing the Community	Assessing local needs and resources
**Theme 2: Identifying Needs**	
** Module 5:** Choosing a Strategy	Deciding on a focus area
** Module 6:** Advocacy Skills	Building capacity for advocacy
** Module 7:** Stakeholder Identification	Identifying and contacting stakeholders
** Module 8:** Asset Mapping	Asset mapping and strength Identification
**Theme 3: Planning for Next Steps**	
** Module 9:** Leadership Skill Building	Leadership development
** Module 10:** Vision Planning	Developing group mission and logic model
** Module 11:** Action Planning	Developing an action plan
** Module 12:** Monitoring and Evaluation	Assessing project outcomes
**Theme 4: Action Part I**	
** Module 13:** Implementation	TBD – based on specific Change Club
** Module 14:** Implementation	TBD – based on specific Change Club
** Module 15:** Implementation	TBD – based on specific Change Club
** Module 16:** Progress Update	TBD – based on specific Change Club
**Theme 5: Action Part II**	
** Module 17:** Implementation	TBD – based on specific Change Club
** Module 18:** Implementation	TBD – based on specific Change Club
** Module 19:** Implementation	TBD – based on specific Change Club
** Module 20:** Progress Update	TBD – based on specific Change Club
**Theme 6: Next Steps**	
** Module 21:** Implementation	TBD – based on specific Change Club
** Module 22:** Implementation	TBD – based on specific Change Club
** Module 23:** Implementation	TBD – based on specific Change Club
** Module 24:** Closing and Wrap-Up	Program Conclusion

The first set of modules include building group rapport and identity and establishing group norms. CC members will engage in online modules outside of meetings that discuss nutrition and PA topics, with a focus on social and environmental barriers and facilitators. During each meeting, facilitators will encourage members to share what they have learned and how they are implementing individual-level change. During the subsequent modules, which focus on issue identification and action planning phases, CCs will conduct an assessment of community assets [84], review a menu of possible built environment changes, and select one or more that can feasibly be implemented in the community within six months. To maximize potential for effectiveness, menu options: 1) are recommended by the Community Preventive Services Task Force [85]; 2) earned a Class I or a Class II rating from the American Heart Association as population approaches to improve diet or PA behavior, indicating the weight of the evidence for the intervention is in favor of efficacy [86]; and/or 3) are recommended by the Global Action Plan for the Prevention and Control of Noncommunicable Diseases [87].

### Participant retention

We will implement multiple common and effective retention strategies, including participation tracking procedures, using multiple contact methods, an accessible phone number for support, keeping in regular contact, highlighting the benefits of research, and using validated surveys [88–93]. We will send notifications via phone, email, text, and/or postal mail to participants at regular intervals, which has worked well to minimize attrition in our prior rural community intervention studies. These notifications may include non-religious holiday (e.g., New Year) or seasonal (e.g., ‘Welcome back, Spring!’) postcards, and messages via email, text, and phone related to upcoming data collection. We have had success retaining participants (80–95% retention) in prior studies with similar populations and timeframes [94].

### Participant compensation

Participants will be compensated $75 at each study timepoint (baseline, 12 months, 24 months, and 36 months) for completing the following: online survey, 24-h dietary recall, and self-reported pedometer or wearable fitness tracker readings. Participants who complete all data collection activities across the four timepoints will be provided an additional bonus at the end of the study ($150 for CCM and $75 for friends and family members and community residents). Some participants will be invited to complete data collection for process evaluation. Additional compensation for those activities is detailed in Table 5. All compensation will be given in the form of an electronic gift card or through a mobile payment app.

### Outcome assessment

Outcome data will be collected via online survey which will include self-measurement of height, weight, and waist circumference; a 24-h dietary recall collected via the Automated Self-Administered 24-h Dietary Assessment Tool (ASA24) [95]; and self-reported pedometer or wearable fitness tracker readings. Survey data will be collected using the Qualtrics application. All data will be coded using participant identification numbers instead of participant names. Only the Principal Investigator and the research staff will have access to the list that matches the names with the participant identification number. Data will be stored in a secure central location and access to files will be restricted to specific study staff. The contents of identifiable data files will be encrypted to secure data. SimpleStep Rechargeable Step Counters (Pedometer Express_,_ Cedar Minnesota, USA) or a wearable fitness tracker owned by the participant (e.g., Fitbit) will be used to obtain objective data on participant PA. The participant-owned fitness tracker must be comparable to the pedometer provided by the project (e.g., 3D motion sensor). Pedometers will be worn for seven days at each time point. Participants will record their daily steps and then report them to attain valid and reliable estimates of participants’ average daily PA.

LS7 score at 24-month follow-up is the primary efficacy endpoint. LS7 is a 7-item composite cardiovascular health score correlated with prevalence of cardiovascular disease events [96, 97]. Each item is classified as poor (0), intermediate (1), or ideal (2) (see Table 3). Scores for each of the seven items are summed for a total LS7 score between 0 and 14, with higher scores indicating better health.

**Table 3:** American Heart Association’s Life’s Simple 7 components and scoring

Indicator	Poor (0)	Intermediate (1)	Ideal (2)
Smoking	current smoker	quit < 12 months ago	never smoked or quit > 12 months ago
BMI	obese (> 30)	overweight (25–29.9)	healthy weight (< 25)
Physical activity	none	some (1–149 min/week of moderate or 1–74 min/week of vigorous)	recommended amount (≥ 150 min/week of moderate or ≥ 75 min/week of vigorous)
Healthy diet indicators met • ≥ 4.5 cups/day of FV • ≥ 2 servings/week of fish • ≥ 3 servings/day of whole grains • ≤ 36 oz/week of sugar‐sweetened beverages • ≤ 1500 mg/day of sodium	0 or 1 indicators	2 or 3 indicators	4 or 5 indicators
Cholesterol	high (≥ 240 mg/dL)	borderline high (200–239 mg/dL) or normal with medication	normal (< 200 mg/dL)
Blood pressure	high (≥ 140 mmHg systolic or ≥ 90 mmHg diastolic) or diagnosed with coronary heart disease, heart attack, heart failure, stroke, vascular disease, or congenital heart defects	elevated (120–139 mmHg systolic or 80–89 mmHg diastolic) or normal with medication	normal (< 120 mmHg systolic and < 80 mmHg diastolic)
Glucose	diabetes (≥ 126 mg/dL)	prediabetes (100–125 mg/dL) or normal with medication	normal (< 100 mg/dL)

Assessment of secondary outcomes will also focus on the 24-month follow-up timepoint. There are 24 secondary outcomes at the individual level, two of which are objective values (see Table 4). In addition, there are six outcomes at the community/collective level (e.g., social cohesion, community investment, civic engagement) and six outcomes at the environmental level (e.g., neighborhood safety, food availability, walking environment), all of which are assessed with tools adapted from validated instruments.

**Table 4:** Data collection schedule

Data	Measure	Before baseline	Baseline	+ 12 months	+ 24 months	+ 36 months
Electronic informed consent	Institutional Review Board-approved consent	X				
Sociodemographics	Sociodemographic questions [98]		X			
Adverse event monitoring	Standard monitoring		X	X	X	X
Social determinants of health	Questions adapted from Billioux et al. [99] and Gadhoke et al. [100]		X	X	X	X
Food security	Brief assessment [101]		X	X	X	X
Social network characteristics	Social network questions [102]		X	X	X	X
**Primary outcome—individual**						
LS7 cardiovascular health score (0–14)	Composite [103, 104]		X	X	X	X
**Secondary outcomes—individual**						
BMI^a^	Self-measured		X	X	X	X
Waist circumference (in)	Self-measured		X	X	X	X
High/elevated blood pressure (y/n)^a^	Classified from self-reported measurements, diagnosis, and medication use		X	X	X	X
High/borderline total cholesterol (y/n)^a^	Classified from self-reported measurements, diagnosis, and medication use		X	X	X	X
Diabetes/pre-diabetes (y/n)^a^	Classified from self-reported measurements, diagnosis, and medication use		X	X	X	X
General health status	SF-36 general health item [105]		X	X	X	X
Current smoker (y/n)^a^	LS7 item [103, 104]		X	X	X	X
Total HEI score (1–100)	Single 24-h recall collected via the ASA24 [95]		X	X	X	X
Total fruit and vegetable intake (cups/day)^a^	LS7 item [103, 104]; ASA24 [95]		X	X	X	X
Consumption of whole grains (servings/day)^a^	LS7 item [103, 104]; ASA24 [95]		X	X	X	X
Fiber intake (g/day)	DSQ [106, 107]; ASA24 [95]		X	X	X	X
Met recommendation for fish consumption (y/n)^a^	LS7 item [103, 104]		X	X	X	X
Consumption of ultra-processed foods (% total kcal)	ASA24 [95]		X	X	X	X
Frequency of consuming ultra-processed foods (times/month)	Adapted from DSQ [106] and BSQ2 (9 items) [108]; ASA24 [95]		X	X	X	X
Red and processed meat consumption (g/week)	Estimated from DSQ [106, 109]; ASA24 [95]		X	X	X	X
Alcohol consumption (drinks/day)	Adapted from the AUDIT (2 items) [110]; ASA24 [95]		X	X	X	X
Total steps per day	Average of self-reported pedometer or wearable fitness tracker readings		X	X	X	X
Total physical activity (MET-min/week)^a^	IPAQ-long [111]		X	X	X	X
World Cancer Research Fund/American Institute for Cancer Research recommendation adherence score (0–7)	Composite [112]		X	X	X	X
Healthy eating motivation (1–5)	Adapted Naughton & McCarthy Healthy Eating Motivation Scale (3-items) [113]		X	X	X	X
Confidence for healthy eating (1–5)	Adapted from Sallis Eating Habits Confidence Survey and Seguin-Fowler Expanded Eating Habits Confidence Survey (7-item) [114, 115]		X	X	X	X
Social support for healthy eating (1–5)	Ball Social Support for Healthy Eating Scale [116]		X	X	X	X
Exercise attitudes (1–5)	Adapted from Sect. 2, question 5 of the AARP Exercise Attitudes and Behaviors Survey (4 items) [117]		X	X	X	X
Exercise confidence (1–5)	Adapted from Sallis Exercise Confidence Survey (3 items) [115]		X	X	X	X
Social support for physical activity (1–5)	Ball Social Support for Physical Activity Scale [116]		X	X	X	X
**Secondary outcomes—collective**						
Social engagement (family and friends) (1–5)	Lubben Social Network Scale [118]		X	X	X	X
(Community) social cohesion (1–5)	Social cohesion sub-scale of the Mujahid et al. NES [119]		X	X	X	X
Individual mobilization (1–5)	Human capital sub-scale of the Jakes & Shannon Mobilization Scale – Individual [120]		X	X	X	X
General civic engagement attitudes (1–5)	Attitudes sub-scales of the Doolittle & Faul Civic Engagement Scale [121]		X	X	X	X
General civic engagement behaviors (1–5)	Behaviors sub-scale of the Doolittle & Faul Civic Engagement Scale [121]		X	X	X	X
Investment in community health (number of priorities) (0–5)	Investment in community health sub-scale of the RWJF National Survey of Health Attitudes [122]		X	X	X	X
**Secondary outcomes—environment**						
Walking environment (1–5)	Adapted walking sub-scale of the NES (7 items) [119]		X	X	X	X
Neighborhood safety (1–5)	Adapted neighborhood safety sub-scale of the NES (3 items) [119]		X	X	X	X
Neighborhood aesthetic (1–5)	Adapted neighborhood aesthetic sub-scale of the NES (2 items) [119]		X	X	X	X
Fresh FV availability (1–5)	Adapted from fresh fruit and vegetable availability sub-scale of the Green & Glanz NEMS-P (3 items) [123]		X	X	X	X
Store selection motivation (1–5)	Adapted from the store selection motivation sub-scale of NEMS-P (3 items) [123]		X	X	X	X
Restaurant healthy food availability (1–5)	Adapted from the restaurant healthy food availability sub-scale of NEMS-P (2 items) [123]		X	X	X	X

^a^Simple 7 components

*ASA24* Automated Self-Administered 24-Hour Dietary Assessment, *AUDIT* Alcohol Use Disorders Screening Test, *BMI* body mass index, *BSQ* Beverage and Snack Questionnaire, *DSQ* NHANES Dietary Screener Questionnaire, *HEI* Healthy Eating Index, *IPAQ-long* International Physical Activity Questionnaire long form, *LS7* American Heart Association’s Life’s Simple 7, *SF-36* 36-Item Short Form Survey, *NEMS-P* Perceived Nutrition Environment Measures Survey, *NES* Neighborhood Environment Scale, *RWJF* Robert Wood Johnson Foundation

### Process evaluation

The process evaluation is designed to understand implementation of both the diet and PA content and the civic engagement aspect of the intervention. We will assess implementation outcomes (see Table 5): dose received (acceptability and appropriateness of the intervention, how participants experienced the intervention, attendance, satisfaction, cultural compatibility/relevance); fidelity (to what degree the intervention was implemented as intended, what was adapted and how); feasibility (perceptions on how feasible it was to integrate the intervention into usual activities); and group functioning (functional and dysfunctional group dynamics, satisfaction with the group) [124]. Using the Consolidated Framework for Implementation Research (CFIR) [125], we will also collect data related to barriers and facilitators to implementation that could impact future uptake of the intervention. The CFIR has 26 constructs within five major domains: intervention, inner and outer settings, individuals involved, and process by which implementation is accomplished. The study team has pre-selected the constructs most relevant to implementation of both the nutrition and PA content and civic engagement component and are the most likely to vary across community sites. Lastly, process evaluation data will help elucidate CC action plan outcomes (goal achievement and impact) as well as the mechanisms by which any individual-level outcomes are realized among CCM, friends and family members, and community residents. Data will be collected using both surveys and qualitative in-depth interviews. More details about the process evaluation data collection instruments and their purpose and time schedule are presented in Table 5.

**Table 5:** Process evaluation assessment plan and timeline

INSTRUMENT	DATA COLLECTED	PARTICIPANT CATEGORY	TIMEPOINT(S)	COMPENSATION	NOTES
Post-Meeting Surveys	-Implementation outcomes, nutrition and PA content (*dose received*: attendance, perceived participant satisfaction, engagement, appropriateness/fit, relevance/usefulness; *fidelity*: components covered, to what degree, enhancements/modifications, meeting start & end times; *feasibility*: prep time, cost)	Facilitators/ Educators	After each meeting	N/A	CC cost questions asked on final Post-Meeting Survey
Post-Theme Surveys	-Implementation outcomes, nutrition & PA content (dose received) -Implementation outcomes, CC action plan (group functioning) -Mechanisms of outcomes (perceptions about personal behavior change)	CCM^a^	After each curriculum theme^b^	$20^c^	Participant cost questions asked on first Post-Theme Survey only
Post-Curriculum Interview Guide	-Barriers and facilitators to implementation of nutrition and PA content (CFIR constructs)	Educators	At conclusion of all curriculum modules	N/A	
Post-Curriculum Interview Guide	-Implementation outcomes, nutrition & PA content (dose received) -Implementation outcomes, CC action plan (group functioning) -Mechanisms of outcomes (perceptions about personal behavior change; perceptions about how CC action plan will impact community)	CCM^d^	At conclusion of all curriculum modules	$40	
Check-In Survey	-Implementation outcomes of the CC action plan (fidelity to action plan, group functioning) -CC action plan outcomes (goal achievement, impact)	Educators	Monthly for 12 months, starting one month after conclusion of all curriculum modules	N/A	CC cost questions asked on final Check-In Survey
One Year Post Interview Guide	-Barriers and facilitators to implementation of the CC action plans (CFIR constructs) -CC action plan implementation outcomes (fidelity, group functioning) -CC action plan outcomes (goal achievement, impact) -Mechanisms of outcomes (CCM only: perceptions about personal behavior change)	Educators, CCM	12 months after the conclusion of all curriculum modules	$40 (CCM only)	
Mini-Interview Guide	-CC action plan outcomes (perceptions of impact on community) -Mechanisms of community resident outcomes (perceptions of impact on self)	Community Residents^e^	12 months after the conclusion of all curriculum modules	$20	
Online Survey: Process Evaluation Questions	-CC action plan outcomes (current activities, goal achievement, new initiatives)	CCM	12-, 24-, and 36-month timepoints	Part of overall study compensation	Process evaluation questions will be included in the annual online survey
Online Survey: Process Evaluation Questions	-Mechanisms of friends and family members, community resident outcomes (awareness of CC project^a^; involvement in CC project^a^; involvement in any other community change initiatives; awareness of changes in behavior or weight loss of community members)	Friends and Family Members, Community Residents	12-, 24-, and 36-month timepoints	Part of overall study compensation	Process evaluation questions will be included in the annual online survey
Two Year Post Interview Guide	-CC action plan outcomes (goal achievement, new initiatives) -Implementation outcomes (sustainability)	Educators	24 months after the conclusion of all curriculum modules	N/A	Will include CC cost questions

^a^ Only those in intervention community

^b^ Or at other appropriate intervals, depending on meeting structure

^c^ Must complete all surveys to receive compensation

^d^ Random sub-sample from each CC

^e^ Approximately 15 per intervention community

*CC* Change Club, *CCM* Change Club Members, *CFIR* Consolidated Framework for Implementation Research, *PA* Physical Activity

Qualitative interviews will be analyzed using a directed qualitative content analysis approach [126–128] in a two-step process: an initial codebook will be drafted based upon the questioning structure; transcripts will then be reviewed, and codes added for topics that arise consistent with the formative evaluation goals. The NVivo program (QSR International) will be used to assist with data analysis. Transcripts will be coded so that comparisons can be made across communities. Once the coding scheme is finalized, a sample of 10% of randomly chosen transcripts will be coded by two team members to estimate inter-rater reliability [126]. Code definitions will be refined until high inter-rater reliability is achieved. The remaining transcripts will be coded using this final codebook with systematic checks for fidelity. Coded text will then be summarized by team members to highlight key themes and sub-themes. We will use these codes to identify themes by location, key distinctions in experiences, relationships with quantitative data, and important non-conforming cases.

### Cost analysis

Cost analysis will include a program and site-specific cost analysis as well as an exploratory cost-effectiveness analysis on LS7 (and secondary outcomes as appropriate) from the payer perspective, which means we will estimate how much the intervention’s payer or sponsor paid for the intervention, focusing on costs directly incurred to administer and implement the program. We will collect data on the costs of the resources used in CC interventions at approximately 6-, 18-, and 30-months post-CC initiation. The costs incurred by CCM, such as travel and equipment in order to participate, will be based on costs spent to attend the first meeting. Collection of cost data will be integrated into the process evaluation surveys of CCM and facilitators. Costs will include wages for CC facilitator time and rented meeting space. Other costs will vary across the menu of CC interventions (e.g., resources used in media and educational campaigns, trail signage). Total costs for each CC will be summed and compared with CC impact estimates in each community.

### Social network analysis

We will use egocentric social network analysis to assess outcomes among CCMs and their friends and family, and whether social network properties, including composition and structure, are associated with outcomes among participants. Egocentric networks will be identified using the unique link each CCM uses to invite their friends and family to participate, and name interpreter and inter-relater questions will be included in the surveys completed by CCMs and their friends and family members [129]. CCM members will then answer name interpreter questions about their friends and family members including how often they see each friend or family member and the support they receive from each friend or family member. Friends and family members also will respond to identical questions about the CCM. Compositional and structural variables will be calculated using E-NET statistical software [130], and analyses will be completed using R statistical language software [131].

### Statistical analysis

#### Sample size determination

Our sample size targets are based upon the ability to detect significant difference between arms at the 24-month post-baseline time period using a t-test, with a desired power of 80%, an alpha level of 0.05, and a two-sided test. The sample size requirements for this study are based on our prior intervention studies [132, 133], which were six months in length and demonstrated an effect size of + 0.7–0.8 units in the LS7 composite score. We aim to recruit 70 CCM in intervention and 70 in control and, based on our prior CC work [133], we estimate 10% attrition each year, resulting in 57 CCM in each arm at 24-month follow-up. Cluster sizes were assumed to be consistent across communities, and the intraclass correlation of participants within those clusters is estimated to be 0.025. This adjustment results in an effective sample size of 45 CCM per arm and an ability to detect an effect size of 0.60 SDs (approximately 1 unit in the LS7 composite score).

We will enroll 560 friends and family members in intervention communities and 560 in control communities but anticipate greater attrition (20% per year) and larger clustering of friends and family members around CCM and within communities (ICC = 0.08). The effective sample size will be 252 friends and family members per arm at 24-month follow-up, and an ability to detect an effect size of 0.25 (approximately 0.4 change in LS7 composite score).

We also will enroll 400–500 community residents in intervention communities and 400–500 in control communities. We estimate 20% attrition per year among community residents and an ICC of 0.025, resulting in an ability to detect an effect size of 0.36 (mid-way between the effect sizes detectable in CCM and friends and family members samples).

#### Frequency of data review

The frequency of data review is as follows: 1) participant enrollment will be reviewed monthly; 2) adverse events will be reviewed as they occur; 3) stopping rules regarding statistical power implications of dropouts and missing data will be reviewed yearly.

#### Outcome analysis

All continuous variables will be examined for normality and, if not met, outliers will be investigated and rectified, and variables will be transformed to improve the distributions or non-parametric tests will be used [134]. Univariate descriptive statistics for all variables will be examined for all sample members pooled. Missing data will be examined for missingness at random and imputed using hierarchical multiple imputation methods [135–137]. Baseline outcome measures will be summarized with means and standard deviations for each treatment arm separately. Comparisons across arms will be completed using Chi-square tests (binary and categorical variables), t-tests (continuous variables), or non-parametric tests when warranted. Change from baseline will be calculated for each outcome variable at each point in time. Change at 36 months relative to 24 months also will be calculated to examine behavior maintenance.

Mixed models will test for differences in change in outcomes 24 months post-baseline between intervention and control arms by modeling a change score with control for baseline value and including community as a random effect to reflect clustering of persons within community (the level at which random assignment will occur). For dichotomous outcomes (e.g., current smoker, met whole grain recommendation), variables will be analyzed as assessed at 24 months post baseline with control for baseline value, and community as random effect. Secondary endpoints analysis will be conducted similarly to test for 12-month and 36-month change from baseline in intervention relative to control, as well as 36-month change from 24-month to test for maintenance of any observed 24-month effects. All tests will be considered significant at p < 0.05 with two-tailed tests.

#### Dissemination of project findings

Dissemination will include multiple sectors in line with the goals of the CEBEC intervention. Results will be shared with community members, extension and community organization stakeholders, policymakers, and researchers. We will provide information on results to community members through articles in lay press (e.g., newspapers), community meetings, and on the study website. We will disseminate results to organization staff and leadership through lunchtime meetings/presentations and summary sheets. We will develop materials that are tailored to policymakers (e.g., briefs using graphics to summarize key findings), and reach researchers by publishing in peer-reviewed practice-oriented journals, including open-access journals, and deliver presentations at scientific meetings. We will share study resources and data through a resource and data sharing agreement. The PI will have access to the final trial dataset; data are available for use by others via request to the PI.

## Discussion

Evidence demonstrating positive change in behaviors and health outcomes following CEBEC interventions is limited in scope but shows notable and encouraging outcomes (e.g., increased PA) [60, 63–67]. Therefore, the opportunity to collect these data prior to implementation and throughout a follow-up period, and to do so with an adequate number of comparison communities, presents a critical and innovative research opportunity with the potential to identify effective and cost-effective strategies for dissemination. Thus, the central hypothesis is that the CC, a CEBEC intervention approach, will improve health behaviors and outcomes among engaged residents and their friends and family, and that these changes can catalyze critical steps in the pathway to improving rural health equity through improved healthy eating and PA opportunities.

## Supplementary Information

**Additional file 1.**

## Data Availability

Data sharing is not applicable to this article as no datasets have been generated or analyzed.

## Abbreviations

AARP: American Association of Retired Persons
ASA24: Automated Self-Administered 24-Hour Dietary Assessment
AUDIT: Alcohol Use Disorders Screening Test
BMI: Body Mass Index
BSQ2: Beverage and Snack Questionnaire version 2
CC: Change Club
CCM: Change Club Member
CEBEC: Civic Engagement for Built Environment Change
CFIR: Consolidated Framework Implementation Research
DSQ: NHANES Dietary Screener Questionnaire
FV: Fruit and Vegetables
HEI: Healthy Eating Index
ICC: Intra-class Correlation Coefficient
IPAQ-long: International Physical Activity Questionnaire long version
LS7: American Heart Association’s Life’s Simple 7
MET: Metabolic Equivalent of Task
NCI: National Cancer Institute
NEMS-P: Perceived Nutrition Environment Measures Survey
NES: Neighborhood Environment Scale
PA: Physical Activity
RWJF: Robert Wood Johnson Foundation
SF-36: 36-Item Short Form Survey
